# Heterologous expression of heat stress-responsive AtPLC9 confers heat tolerance in transgenic rice

**DOI:** 10.1186/s12870-020-02709-5

**Published:** 2020-11-11

**Authors:** Yuliang Liu, Xinye Liu, Xue Wang, Kang Gao, Weiwei Qi, Huimin Ren, Haorui Hu, Daye Sun, Jiaoteng Bai, Shuzhi Zheng

**Affiliations:** 1 Ministry of Education Key Laboratory of Molecular and Cellular Biology, Hebei Collaboration Innovation Center for Cell Signaling, Hebei Key Laboratory of Molecular and Cellular Biology, College of Life Sciences, Hebei Normal University, Shijiazhuang, 050024 China; 2 Shijiazhuang No.1 High School, Pingan North Street, Shijiazhuang, 050010 China; 3 South 2nd ring east road 20, Hebei Key Laboratory of Molecular and Cellular Biology, Hebei Normal University, Shijiazhuang, 050016 China

**Keywords:** AtPLC9, Ca^2 +^, Heterologous expression, Rice, Thermotolerance

## Abstract

**Background:**

As global warming becomes increasingly severe, it is urgent that we enhance the heat tolerance of crops. We previously reported that *Arabidopsis thaliana* PHOSPHOINOSITIDE-SPECIFIC PHOSPHOLIPASE C9 (AtPLC9) promotes heat tolerance.

**Results:**

In this study, we ectopically expressed *AtPLC9* in rice to examine its potential to improve heat tolerance in this important crop. Whereas AtPLC9 did not improve rice tolerance to salt, drought or cold, transgenic rice did exhibit greater heat tolerance than the wild type. High-throughput RNA-seq revealed extensive and dynamic transcriptome reprofiling in transgenic plants after heat stress. Moreover, the expression of some transcription factors and calcium ion-related genes showed specific upregulation in transgenic rice after heat stress, which might contribute to the enhanced heat tolerance.

**Conclusions:**

This study provides preliminary guidance for using AtPLC9 to improve heat tolerance in cereal crops and, more broadly, highlights that heterologous transformation can assist with molecular breeding.

**Supplementary Information:**

**Supplementary information** accompanies this paper at 10.1186/s12870-020-02709-5.

## Background

Plants must cope with a complicated and varied environment. The primary abiotic stresses plants facing include heat, cold, drought and salt. Temperature affects plant growth, development, and geographical distribution, and extreme temperatures can adversely affect crop quality and productivity. As temperatures increase above the optimum, plants begin to experience heat stress (HS). Plants perceive and transmit HS signals through a complicated pathway. Heat stress initially increases the fluidity of the plasma membrane, and researchers have proposed that HS activates cyclic nucleotide-gated channel family proteins through these changes in membrane fluidity [1, 2]. While it is still not clear what the HS signal transduction comprises, a number of HS signal components capable of regulating thermotolerance in plants have been identified, including Ca^2+^, calcium-dependent protein kinases (CDPKs), 1,4,5-inositol triphosphate (1,4,5-IP_3_), cyclic AMP (cAMP) and mitogen-activated protein kinases (MPK) [3, 4]. Moreover, gene expression changes are critical for plant adaptation to high-temperature stress. Recent research reports that transcription factors are important for plant responses to high temperature at the transcription level [5]. The latest report showed that high temperature-induced stomatal pores opening to facilitate leaf cooling requires blue light, suggesting the existence of an additional intracellular high-temperature response pathway in plants [6].

Phosphoinositide-specific phospholipase C (PLC) is a key enzyme in the phosphatidylinositol signalling system. Many species have multiple versions of PLC, and *Arabidopsis thaliana* has nine isoforms [7]. PLCs are involved in plant development and stresses responding. AtPLC2 in *Arabidopsis thaliana* regulates auxin levels in the vegetative and floral organs to influence the development of male and female gametophytes [8]. AtPLC2 also enhances tolerance to endoplasmic reticulum and drought stresses by regulating the biosynthesis of phosphatidic acid, salicylic acid and jasmonic acid [9, 10]. The expression of *TaPLC1* and *TaPLC2* in wheat is induced by salt or drought [11]. *Arabidopsis* PLC3 participates in ABA responses in seed germination and stomatal closure. Seeds of the *atplc3* mutant germinate slowly and are less sensitive to ABA, preventing germination [12]. In addition, prior research in our lab has shown that overexpression of *AtPLC9* and *AtPLC*3 in *Arabidopsis* substantially enhances heat tolerance [13, 14]. The rice genome includes four PLC genes, *OsPLC1*, *OsPLC2*, *OsPLC3* and *OsPLC4*, on chromosomes 3, 5, 7 and 12, respectively. However, *Arabidopsis* and rice *PLC* family genes do not show a high degree of similarity, indicating that the genes have diverged during evolution [15]. OsPLC1 improves salt tolerance in rice [16]. In response to salt stress, OsPLC1 moves from the cytoplasm to the plasma membrane, where it increases cytosolic Ca^2+^, which controls sodium accumulation in the leaves, leading to improved salt tolerance [16]. OsPLC4 also enhances osmotic stress responses [17].

Rice (*Oryza sativa*) is an important staple food crop that are highly sensitive to high temperature stress at every growth stage such as seed germination, growth, and reproduction, particularly during the reproductive and grain-filling stages [18–21]. High temperatures have been shown to negatively affect rice yield and quality. Extremely high temperatures significantly decrease the grain yield by more than 50%, even causing complete loss of harvest in rice plants [22]. High temperature affects the growth of rice at all stages. During flowering stage, heat stress impairs the pollen germination, anther dehiscence and pollen tube elongation, resulting in pollination failure [23]. Moreover, high temperature affects protein synthesis, transport, folding and assembly processes in rice grains and suppresses of genes expression related to starch biosynthesis [24]. It is necessary to improve the adaptability of rice to cope with the high temperature stress. However, the genetic basis for heat tolerance or adaptability to heat stress in crop plants, including rice, is poorly understood.

Heterologous transformation of genes identified in *Arabidopsis* into monocot crop species such as rice can help elucidate gene function and identify genes capable of conferring adaptive traits, such as heat tolerance, in crops. In this study, we aimed to determine similarities and differences in the transduction of heat-shock signals through PLC in rice and *Arabidopsis* and to determine if *AtPLC9* can be used to enhance the heat tolerance of cultivated rice. We cloned *AtPLC9* and ectopically expressed it in the Asian rice cultivar ‘Changyou No. 1’. Using high-throughput RNA sequencing (RNA-seq), we analysed transcriptome-level changes induced by expression of *AtPLC9* in rice under normal and heat stress conditions. We observed that rice heterologously expressing *AtPLC9* exhibited much higher expression of heat shock factors (OsHSFAs) and calcium ion and calmodulin related genes than wild type plants when exposed to HS. Our results suggest that AtPLC9 may enhance heat tolerance in rice and *Arabidopsis* through similar mechanisms. AtPLC9 conferred heat resistance both in monocots and dicots and these results implied that important elements of the HS response are conserved between monocots and dicots. Our results enhance understanding of the molecular mechanisms of heat-shock response in plants and demonstrate that *AtPLC9* is an important and valuable resource to improve heat resistance in molecular breeding programs. More broadly, this study highlights the potential for heterologous transformation to assist in molecular breeding.

## Results

### Heterologous expression of *AtPLC9* improves heat tolerance in rice

**Fig. 1:**
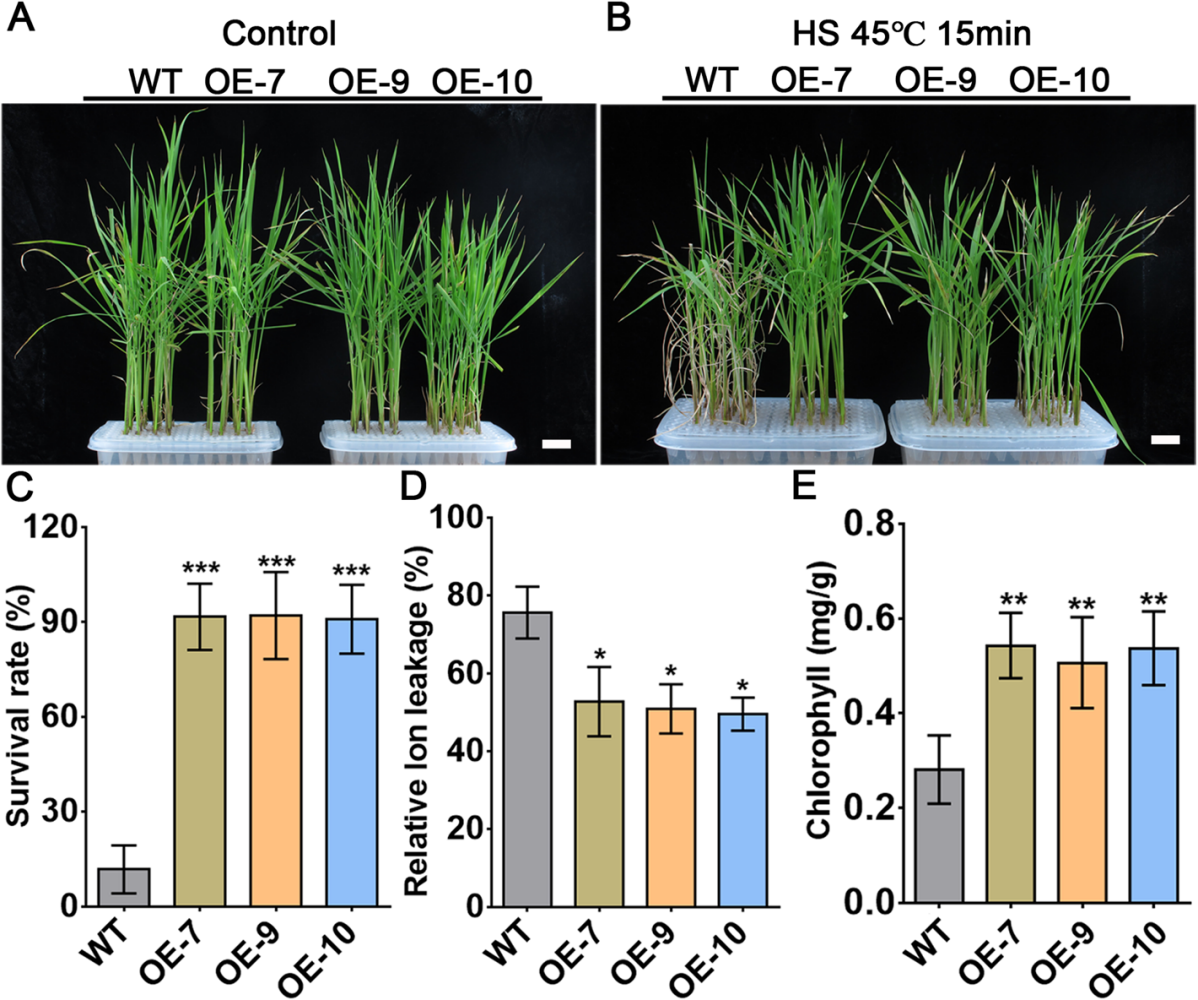
Expression of *AtPLC9* improves the basic tolerance of rice. **a**. Fourteen-day-old rice (WT and transgenic lines OE-7, OE-9 and OE-10) grown in Hoagland’s solution under normal conditions (28 °C) were used as controls. **b**. Seven-day-old rice (WT, OE-7, OE-9 and OE-10) grown in Hoagland’s solution under normal conditions (28 °C) were shifted to 45 °C for 15 min HS treatment and then returned to normal conditions (28 °C) for another 7 days. **c**. Survival rate of WT, OE-7, OE-9 and OE-10 after HS. Each value is the mean ± SE, *n* = 3. **d**. Relative ion leakage of plants after HS treatment. Each value is the mean ± SE, *n* = 3. **e**. Chlorophyll content of seedlings after HS; 0.1 g of fresh tissue was used in each experiment. Each value is the mean ± SE, *n* = 3. Student’s *t*-test was used to calculate *P* values. ***p < 0.001, **p < 0.01, *p < 0.05, statistics was analysed between OE-7/− 9/− 10 vs WT. Bars (**a** and **b**) = 1 cm

To assess the heat-tolerance potential of *AtPLC9* in rice, we heterologously expressed *AtPLC9* coding region driven by 35S promoter, as described in our previous study [14] in ‘Changyou No. 1’ to obtain three independent T4 homozygous transgenic rice lines: OE-7, OE-9 and OE-10. All three lines exhibited higher *AtPLC9* expression levels than the wild type (WT), indicating that they were expressing *AtPLC9* (Supplementary Fig. 1). We then subjected the three lines to HS treatment to examine their phenotypes. Seven-day-old seedlings grown at 28 °C were treated for 15 min at 45 °C or were shifted to 37 °C for 30 min and then returned to normal conditions (28 °C) for 2 h before again subjecting to 45 °C for 45 min to determine basal thermotolerance and acquired thermotolerance, respectively. Seedlings were then grown for another 7 days at 28 °C before we analysed their phenotype, took pictures and calculated their survival rates, electrolyte leakage and chlorophyll content. Significantly more seedlings expressing *AtPLC9* survived compared to the WT in both basal and acquired thermotolerance tests (Fig. 1a, b and Supplementary Fig. 2A, B). WT seedlings exhibited severe wilting and chlorosis and few seedlings survived. In comparison, the three heterologous expression lines had survival rates significantly exceeding that of the WT (Fig. 1c and Supplementary Fig. 2C). Correspondingly, HS-induced electrolyte leakage, an indicator of plasma membrane damage, was dramatically lower in the *AtPLC9*-expressing transgenic plants compared with that in WT plants under HS conditions (Fig. 1d and Supplementary Fig. 2D). The chlorophyll content in three heterologous expression lines was significantly higher than that in WT plants after HS treatment (Fig. 1e and Supplementary Fig. 2E). These results suggest that the heterologous expression of *AtPLC9* greatly improves the heat tolerance of rice.

To examine whether expression of *AtPLC9* confers tolerance to other abiotic stresses, we performed salt, drought and cold stress tests on the transgenic lines. After a 7-day treatment with 200 mM NaCl, the WT and the three transgenic lines all exhibited withered leaves (Supplementary Fig. 3A). All plants died following salt treatment for 14 days, and we observed no difference between WT and the three heterologous expression lines in their response to salt stress (Supplementary Fig. 3B). These results suggest that heterologous expression of *AtPLC9* does not confer enhanced tolerance to salt stress.

We simulated drought stress by treating plants with different concentrations of mannitol. After 2 days growing on 350 mM mannitol, both the WT and the three transgenic lines exhibited withered leaves and were smaller than control plants exposed to 0 mM mannitol (Supplementary Fig. 4A). After 10 days of treatment with 350 mM mannitol, the WT and heterologous expression lines all exhibited dry and withered leaves (Supplementary Fig. 4B). There were no statistically significant differences in survival rate between the WT and the three transgenic lines, suggesting that heterologous expression of *AtPLC9* in rice does not affect drought tolerance.

Previous research has shown that *OsPLDa1* improves the response of rice to cold stress by increasing the cytoplasmic concentration of Ca^2+^, thus increasing the transcriptional activity of COLD-RESPONSIVE C REPEAT/DEHYDRATION-RESPONSIVE ELEMENT BINDING 1 (OsDREB1) [25]. We therefore wondered whether AtPLC9 could also improve the cold tolerance of rice. To address this question, we subjected the WT and the three heterologous expression lines to cold stress. Seedlings grown for 7 days at 28 °C were transferred to 4 °C for 7 days of cold treatment. The WT and heterologous expression lines exhibited similar restrictions in plant height under 4 °C (Supplementary Fig. 5A and 5B), which suggested that AtPLC9 does not affect cold tolerance in rice. In conclusion, the heterologous expression of *AtPLC9* specifically improves the tolerance of rice to heat stress, but not other abiotic stresses.

### Heat stress triggers transcriptome reprogramming in *AtPLC9* heterologous expression lines

To determine how AtPLC9 coordinates the response to HS in rice, we assessed the transcriptome of heterologous expression lines following HS treatment. We first assessed the expression level of heat shock factors (HSFs; *Os03g0745000*, *Os04g0568700*, *Os08g0546800*), Multiprotein-bridging factor 1c (MBF1 *Os06g0592500*) and heat shock proteins (HSPs *OS01g0136000* and *Os03g0266900*). Similar *AtPLC9* expression levels were detected in the three transgenic rice lines (OE-7, OE-9, and OE-10), and they also displayed same phenotype; therefore, only OE-7 was used in subsequent experiments. Before and after HS, none of these genes showed significant differences in their expression levels between OE-7 and WT (Supplementary Fig. 6). This result indicated that AtPLC9 may regulate the HS response through other genes, so we conducted Illumina high-throughput sequencing to search for other regulatory pathways.

**Fig. 2:**
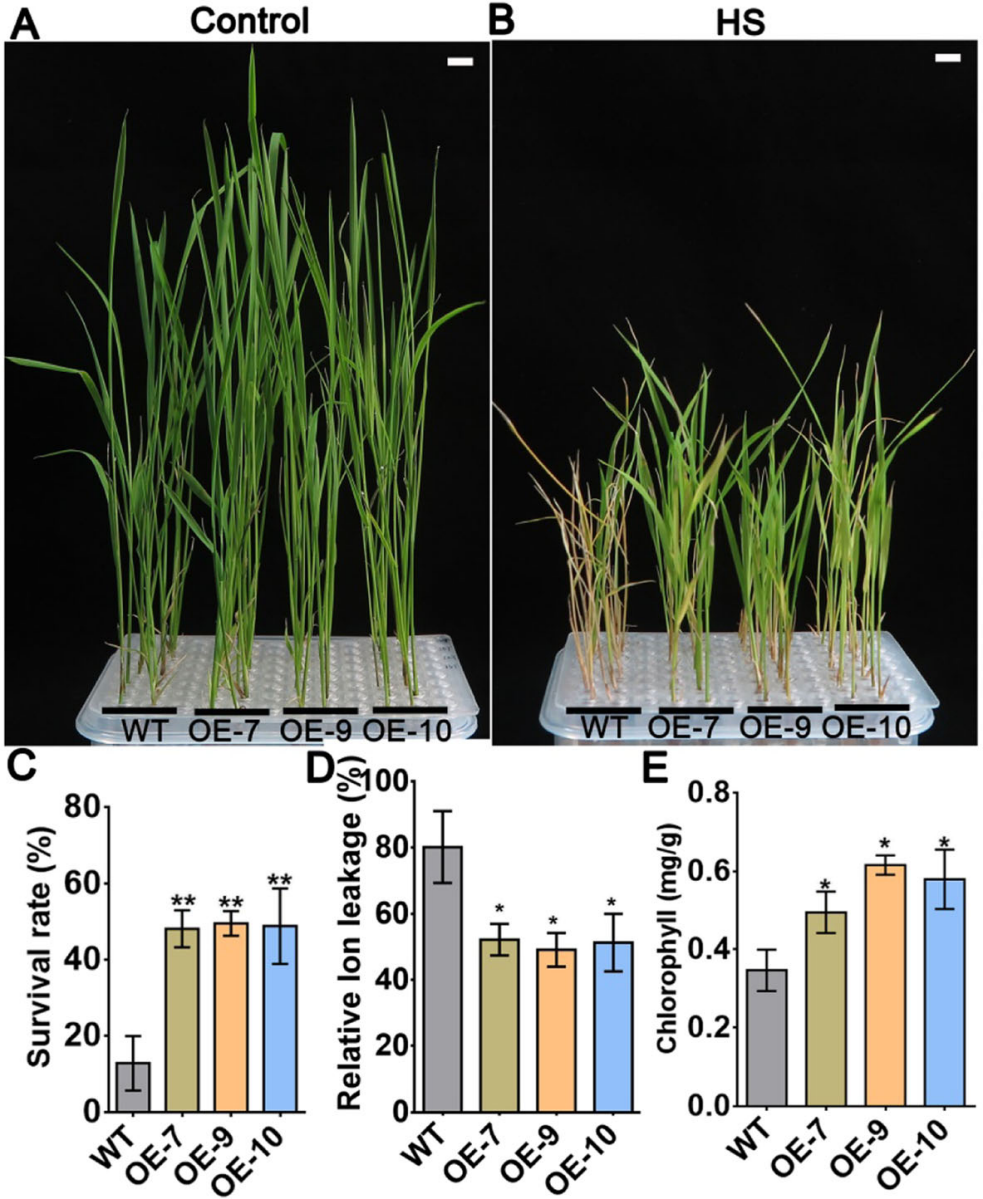
Expression of *AtPLC9* in rice improves acquired thermotolerance. **a**. 14-d-old rice (WT and transgenic lines OE-7, OE-9 and OE-10) grown under normal conditions (28 °C) were used as controls. White bar = 1 cm. **b**. 7-d-old rice (WT, OE-7, OE-9 and OE-10) grown under normal conditions (28 °C) were shifted to 37 °C for 30 min and then returned to normal conditions (28 °C) for 2 h before 45 °C HS treatment for 45 min. After the treatment, the seedlings were returned to 28 °C for another 7 d. White bar = 1 cm. **c**. The survival rate of WT, OE-7, OE-9 and OE-10 after HS. Each value is the mean ± SE, *n* = 3. **d**. Relative ion leakage quantification of plants after HS treatment. Each value is the mean ± SE, *n* = 3. **e**. Chlorophyll content of the seedlings after HS; 0.1 g of fresh tissue was used in each experiment. Each value is the mean ± SE, *n* = 3. Student’s *t*-test was used to calculate the *P* value. ** indicates *P* value < 0.01, * indicates *P* value < 0.05, statistics was analysed between OE-7/− 9/− 10 vs WT

We compared the expression profiles of four samples, designated WT-CK (wild type without HS), WT-HS (wild type after a 15-min HS), OE-7-CK (*AtPCL9*-expressing line 7 without HS) and OE-7-HS (*AtPCL9*-expressing line 7 after a 15-min HS). Each sample contained three biological replicates, from which approximately 879.8 million 150-bp paired-end reads were generated (Supplementary Table 1). After discarding the low-quality reads, 63.6 to 84.2 million reads were kept for subsequent analysis. More than 88% of reads containing no more than three mismatches were mapped to the reference genome, and over 73% of reads showed unique alignments (Supplementary Table 1). Both principal component analysis (PCA) (Fig. 2a) and hierarchical clustering demonstrated high reproducibility among biological replicates (Fig. 2b). Furthermore, PCA revealed a clear divergence in expression profiles between the normal and HS conditions (Fig. 2a). Heat stress amplified the difference in expression profiles between OE-7 and WT (Fig. 2a), which was consistent with our phenotypic observations.

**Fig. 3:**
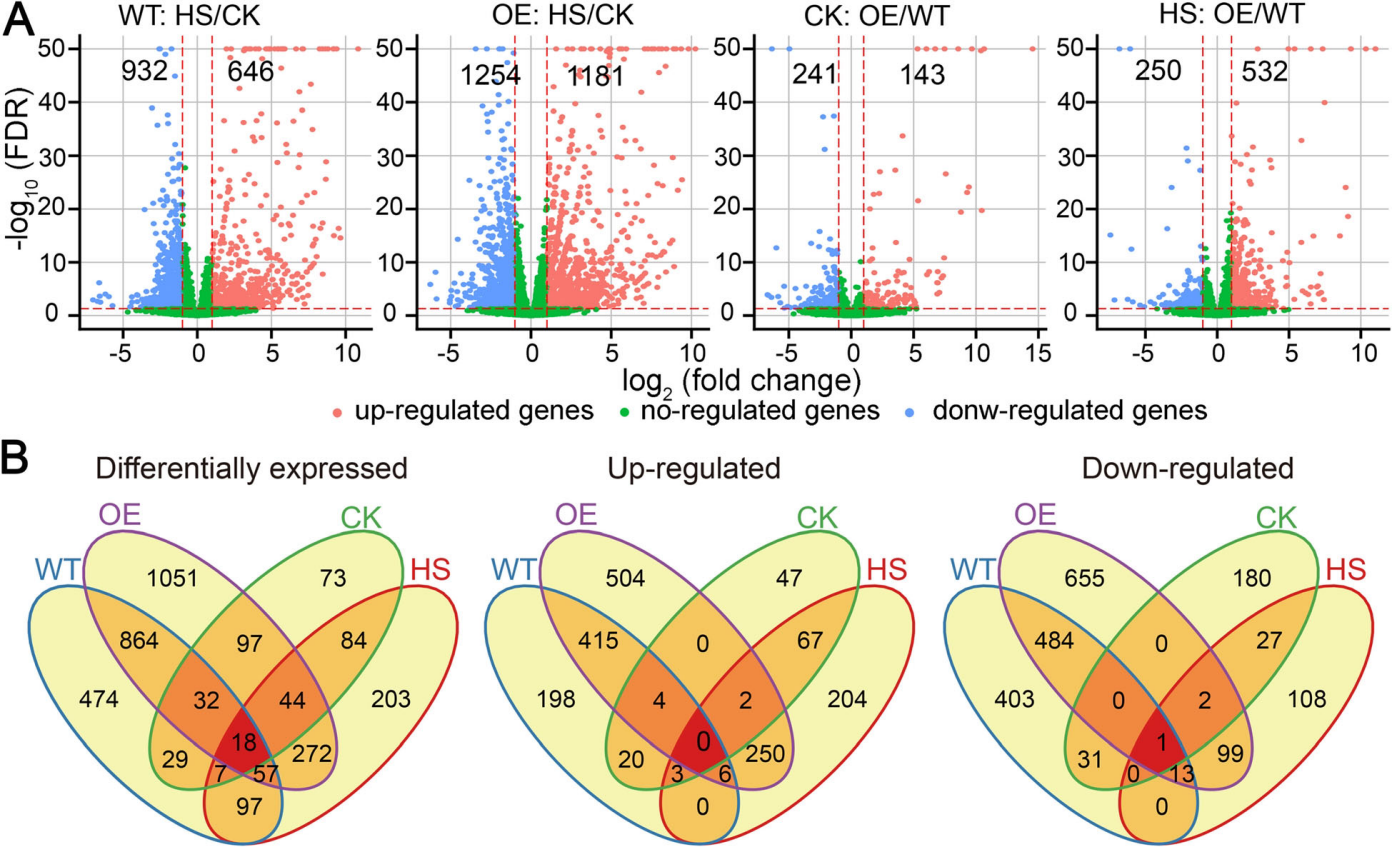
Identification of genes regulated by AtPLC9 during response to heat stress in rice. **a**. Volcano plots of transcriptional changes generated from edgeR. Log2 of fold change is shown on the horizontal axis, and − log10 of the adjusted *P* value (FDR) is shown on the vertical axis. Red and blue dots represent significantly up- and downregulated genes, respectively. Green dots are genes with a nonsignificant change. Vertical and horizontal red dashed lines indicate the cutoff of log2-transformed fold change and -log10 of FDR, respectively. Numbers indicate the counts of genes showing up- and downregulation. **b**. Venn diagram showing the number of differentially expressed (left), upregulated (middle) and downregulated (right) genes in the four comparisons. WT and OE indicate the regulation of gene expression level between heat stress and control in WT and OE-7, respectively. CK and HS denote the change of expression values in OE-7 compared with WT under control and heat stress conditions, respectively

We then performed differential expression analysis between normal and HS conditions for the two genotypes, designated WT:HS/CK and OE-7:HS/CK. Using fold change ≥2 and False Discovery Rate (FDR) < 0.05 as a cutoff, we identified 1578 differentially expressed genes (DEGs) in the WT:HS/CK comparison, of which 932 and 648 genes were upregulated and downregulated by HS, respectively (Fig. 3a; Supplementary Dataset 1). The number of DEGs (2435), upregulated genes (1181) and downregulated genes (1254) in OE-7:HS/CK were all higher than in WT:HS/CK (Fig. 3a; Supplementary Dataset 2), suggesting that more extensive and dynamic transcriptome reprofiling occurred in OE-7 following HS. Venn diagram results revealed that 1464 DEGs (60.12%), 756 upregulated genes (64.01%) and 756 downregulated genes (60.29%) specifically existed in the OE-7:HS/CK comparison (Fig. 3b), which were designated as OE-7-specific genes. We next performed gene ontology (GO) term enrichment analysis of genes showing altered expression levels in WT and OE-7 after HS. Known HS GO terms were significantly enriched among the genes upregulated by heat in both WT and OE-7, including ‘response to heat’ (GO:0009408), ‘protein folding’ (GO:0006457) and ‘response to osmotic stress’ (GO:0006970) (Supplementary Fig. 7). GO terms enriched among genes downregulated in both WT and OE-7 exposed to HS included ‘transport’ (GO:0006810), ‘brassinosteroid homeostasis’ (GO:0010268) and ‘phosphorylation’ (GO:0016310) (Supplementary Fig. 7). Some GO terms were significantly enriched only in the OE-7-specific HS-upregulated genes, such as ‘regulation of response to stress’ (GO:0080134), ‘regulation of metabolic process’ (GO:0019222) and ‘regulation of gene expression’ (GO:0010468) (Supplementary Fig. 7). To further evaluate the overall trend of expression change, we calculated the Pearson correlation coefficient between the fold change of gene expression levels in WT and OE-7 after HS and the fold change of gene expression levels under unstressed conditions. The ratios of expression change for genes related to ‘response to heat’ (GO:0009408) in OE-7 correlated strongly with the corresponding ratios in WT (R = 0.97; Supplementary Fig. 8). However, the expression changes of the remaining genes were only moderately positively correlated (R = 0.58) (Supplementary Fig. 8). These results suggest that the transcriptome reprogramming induced by HS in OE-7 is similar to, but distinguishable from, the corresponding transcriptome changes in the WT. Collectively, the above results suggest that a more extensive and dynamic transcriptome reprofiling occurs in transgenic plants after heat stress, and that this widespread transcriptome change may account for the heat tolerance conferred by heterologous expression of *AtPLC9*.

### Rice heterologously expressing *AtPLC9* under HS exhibits significantly higher expression of transcription factors and Ca^2+^/calmodulin (CaM) signal transduction pathway genes

We also compared the transcriptomes between OE-7 and WT under normal conditions (CK:OE-7/WT) and under HS (HS:OE-7/WT). We detected 384 and 782 DEGs under normal and HS conditions, respectively (Fig. 3a; Supplementary Datasets 3 and 4). Consistent with the PCA (Fig. 2), the number of DEGs under HS was double that under unstressed conditions. Intriguingly, 532 genes showed upregulated expression levels in OE-7 under HS, which was substantially higher than the number of corresponding genes (143) under unstressed conditions (Fig. 3a). However, the number of downregulated genes were similar between the HS (250) and CK (241) comparisons (Fig. 3a). Compared with the CK:OE/WT comparison, we identified 629 DEGs, 460 upregulated genes and 220 downregulated genes specifically existing in the HS:OE/WT comparison (Fig. 3b), representing 80.43, 86.47 and 88.00%. These genes were designated as HS-specific genes. GO term enrichment analysis revealed that those genes upregulated in the HS:OE-7/WT comparison were primarily enriched in the terms ‘regulation of response to stress’ (GO:0080134), ‘regulation of metabolic process’ (GO:0019222) and ‘regulation of gene expression’ (GO:0010468), which were specifically enriched in the OE-7 HS-induced gene set as well (Supplementary Fig. 7).

**Fig. 4:**
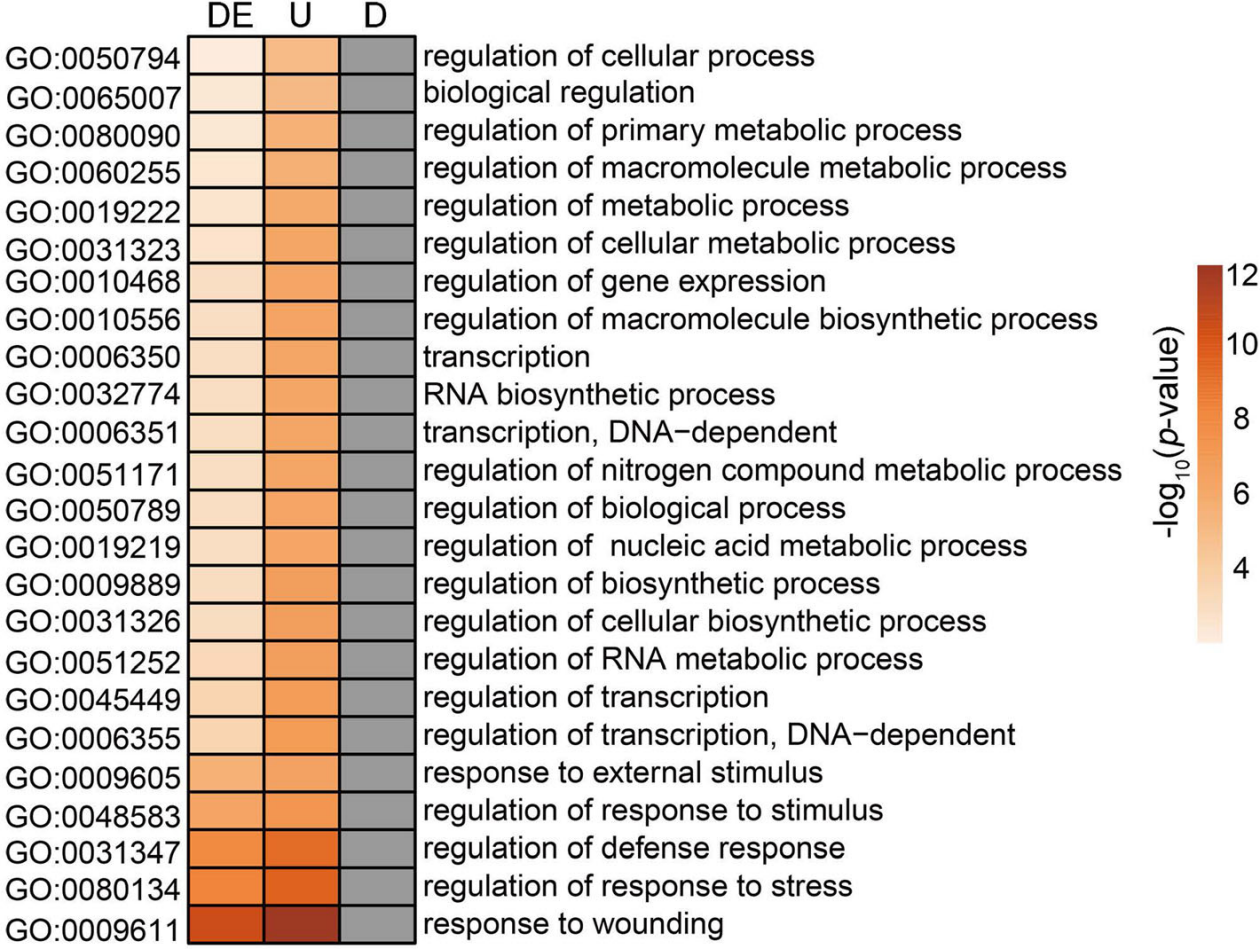
Enriched GO terms among candidate genes regulated by AtPLC9 during response to heat. The colour in each cell indicates -log10-transformed *P* value of the GO enrichment tested by hypergeometric test with Bonferroni correction; blank cells are not significant. DE, U and D indicate differentially expressed, upregulated and downregulated genes, respectively

**Fig. 5:**
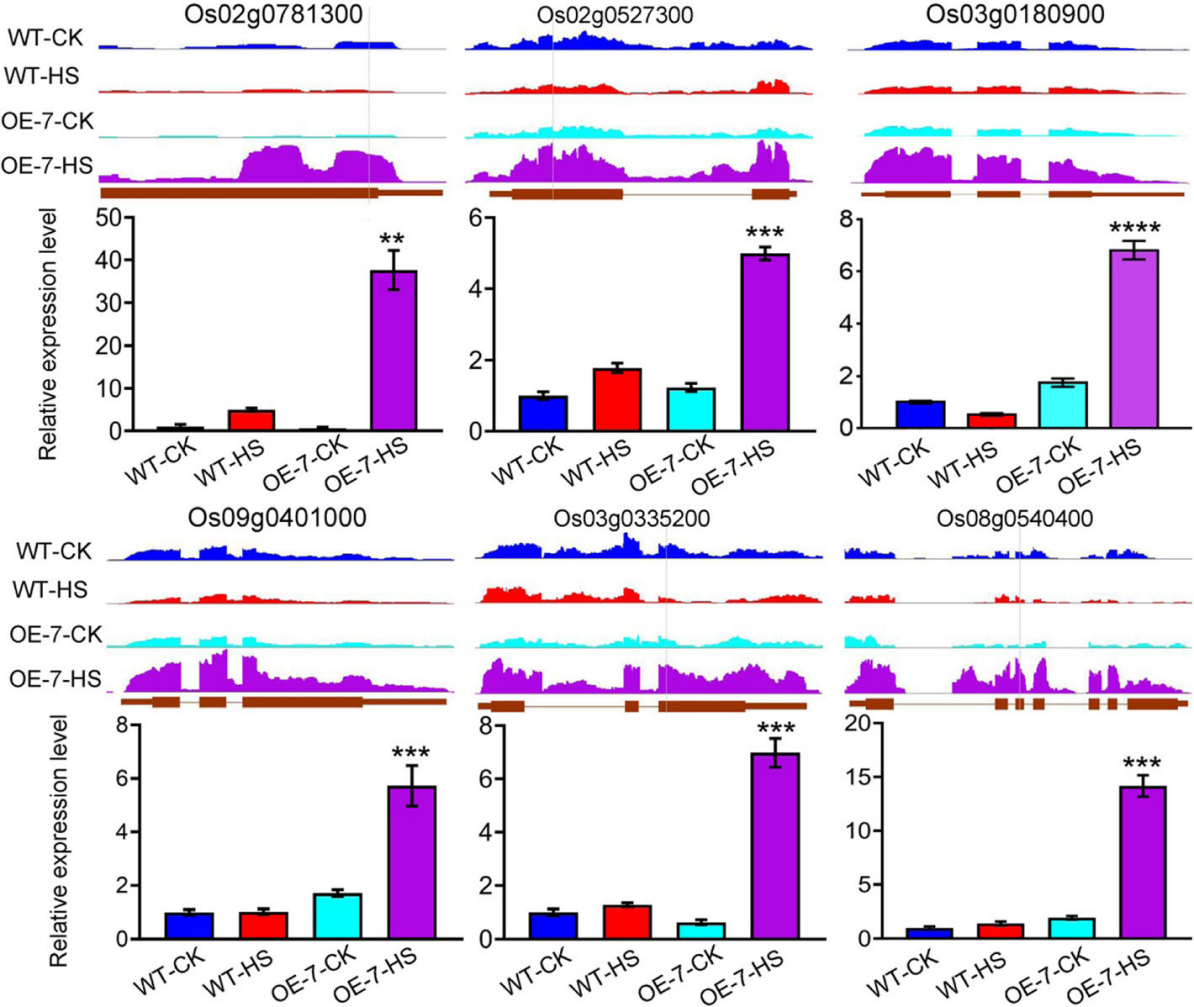
Validation of RNA-seq data using real-time quantitative PCR for six potential AtPLC9 target genes. RNA-seq signals visualized using Integrated Genome Browser (IGB; http://www.bioviz.org) and real-time quantitative PCR quantification of the RNA levels for six candidate AtPLC9-regulated genes in wild-type (WT) and OE-7 plants before (CK) and after HS treatment. Values for RT-qPCR results are means ± SE (*n* = 3). Student’s *t*-test was used to calculate P values. ****P < 0.0001, ***P < 0.001, **P value < 0.01, statistics was analysed between OE-7-HS vs WT-HS

We detected 272 DEGs, 250 upregulated genes and 99 downregulated genes overlapping between OE-7- and HS-specific genes (Fig. 3b), which we identified as the potential targets regulated by AtPLC9 during HS response in rice. We conducted GO term enrichment analysis on these genes to examine the function of targets potentially regulated by AtPLC9 during HS. No specific GO terms were enriched in downregulated target genes (Fig. 4). Upregulated target genes and DEGs were enriched in the same GO terms (Fig. 4). Since AtPLC9 is a positive regulator of the HS response [14], the 250 upregulated target genes were subjected to more detailed analysis (Supplementary Dataset 5). These upregulated target genes were enriched in the GO terms ‘regulation of transcription’ (GO:0045449) and ‘regulation of gene expression’ (GO:0010468) (Fig. 4), which suggested that some of these target genes may be transcription factors (TFs) or transcription regulators (TRs). Using the iTAK pipeline [26], we identified and classified 52 putative TF (51) and TR (1) members from 13 families among these target genes, including WRKY (11; *Os03g0335200*), AP2/ERF (9; *Os02g0781300*), Tify (7; *Os03g0180900*), MYB (6; *Os09g0401000*) and HSF (1; *Os02g0527300*). The Ca^2+^/calmodulin (CaM) signal transduction pathway has a significant role in the heat stress response [14]. From these 250 upregulated target genes, we identified 17 genes annotated as functioning in this pathway, such as calcium-dependent protein kinases (CDPKs). To test the accuracy of our transcriptome-level analysis, we used quantitative RT-PCR to validate the expression levels of six randomly selected upregulated target genes, including five TFs and one CDPK (*Os08g0540400*). Consistent with the RNA-seq data, RT-qPCR revealed that the expression levels of these six genes were substantially elevated in OE-7, OE-9 and OE-10 compared with those in the WT after HS (Fig. 5 and Supplementary Fig. 9). Together, these finding suggest that the expression of particular TFs and Ca^2+^/ CaM pathway-related genes was specifically upregulated by HS in the *AtPLC9*-expressing lines, which may contribute to the improved heat stress tolerance observed in these lines.

## Discussion

In this study, we transformed the *Arabidopsis* gene *AtPLC9* into the rice cultivar ‘Changyou No. 1’, identified its heat-tolerant phenotype, and analysed the transcriptome-level changes induced by *AtPLC9* under normal and HS conditions. This study contributes to our understanding of the biological function, molecular mechanism and heat-tolerance mechanism of AtPLC9 in monocotyledons as well as dicotyledons. We conclude that *AtPLC9* is a valuable gene resource conferring heat resistance both in monocots and dicots, setting the stage for its use to improve crop heat tolerance via molecular breeding programs.

### AtPLC9 confers heat tolerance to monocotyledons as well as dicotyledons

Our previous studies have shown that AtPLC9 and AtPLC3 both enhance heat tolerance in *Arabidopsis thaliana* [13, 14, 27]. In this study, we observed that rice heterologously expressing *AtPLC9* also exhibits improved heat tolerance. The survival rate of *AtPLC9*-expressing lines exposed to 45 °C was eight times more than that of the WT. These results suggest that components of the HS response involving AtPLC9 are conserved between monocotyledons and dicotyledons.

When rice is exposed to salt stress, OsPLC1 moves from the cytoplasm to the cell membrane where it regulates Ca^2+^ responses [16]. In this study, expression of *OsPLC1* and *OsPLC2* was increased after HS treatment. However, *OsPLC3* and *OsPLC4* were not significantly upregulated by heat stress (Supplementary Fig. 10). More research is necessary to identify the molecular mechanisms underlying how OsPLCs respond to HS. Assessing how endogenous OsPLCs function after HS will help us to more fully understand PLC regulation of thermotolerance in rice.

Based on our previous research, we proposed that plants respond to HS through the calcium-calmodulin heat-shock pathway. The calcium-calmodulin pathway is now recognized as a major channel for heat-shock response [2, 28, 29]. Following heat shock, a signal is conveyed from the cell membrane to intracellular targets. Heat shock increases the concentration of cytoplasmic calcium, which in turn increases the expression of CALMODULIN 3 (CaM3). CAM-BINDING PROTEIN KINASE 3 (CBK3) regulates the phosphorylation status and binding activity of heat shock factors (HSFs) to heat shock elements (HSEs), which ultimately regulates thermotolerance by promoting or inhibiting the expression of HSPs [3, 4, 30–32]. In animal cells, IP_3_ binds to the IP_3_ receptor on the endomembrane system, which releases Ca^2+^ from intracellular stores [33, 34]. However, it is not clear whether PLC in plants functions as it does in animals. Preliminary studies have shown that within 1 min following heat shock, the level of IP_3_ rapidly increases. After 3 min, IP_3_ levels reach their maximum of 2.5 times the level prior to heat shock [22]. In addition, IP_3_ can induce the expression of *HSP18.2 promoter: GUS* in the absence of heat shock [3]*.* The phospholipase C-specific inhibitor U73122 limits the heat-shock-induced increase in IP_3_ and Ca^2+^ in *Arabidopsis thaliana* suspension cells [3]. AtPLC9 generates IP_3_ in the cell following HS, stimulating the release of Ca^2+^ from intracellular stores and promoting the transmission of heat-shock signals [14].

In our current study, we observed that rice heterologously expressing *AtPLC9* exhibited much higher expression of OsHSFAs and calcium ion and calmodulin related genes than the WT when exposed to HS. These results suggest that AtPLC9 may enhance heat tolerance in rice and *Arabidopsis* through similar mechanisms and imply that important elements of the HS response are conserved between monocots and dicots.

### AtPLC9 specifically confers tolerance to heat shock, but not to other abiotic stresses

*AtPLC4*, *5*, and *7* in *Arabidopsis* are upregulated under salt or drought stress [35]. Recent research has shown that OsPLC1 regulates salt tolerance in rice by increasing the cytoplasmic concentration of Ca^2+^. Following salt stress, OsPLC1 moves from the cytoplasm to the plasma membrane, where it increases cytoplasmic Ca^2+^, which regulates Na^+^ accumulation in leaves and improves salt tolerance [16]. Prior research in our laboratory indicates that AtPLC9 regulates the concentration of cytoplasmic Ca^2+^ through the second messenger IP_3_ to influence the expression of HSF and HSP and enhance the heat tolerance of *Arabidopsis* [13, 14]. AtPLC4 responds to salt stress, and its mutant also displays a hyposensitivity phenotype [36].

In our current study, we exposed rice heterologously expressing *AtPLC9* to salt and drought stress and observed no significant difference in growth, phenotype or survival rate between transgenic lines and the WT. These results suggest that AtPLC9 functions differently from OsPLCs in rice, and that the former does not affect the sensitivity of rice to salt or drought. Prior research has shown that OsPLDαl responds to cold stress [25]. Yet, after cold stress treatment, we observed no difference in growth between the WT and rice heterologously expressing *AtPLC9*, suggesting that AtPLC9 cannot improve cold tolerance in rice. We conclude that heterologously expressing *AtPLC9* only confers greater tolerance to heat, but not to other abiotic stresses. Cross tolerance is a critical strategy for plant to adapt environmental stress. For example, heat stress tolerance also improves other stress in *Arabidopsis* [37]. However, in this study, we found that AtPLC9 transgenic rice specifically response to heat stress (Fig. 1 and Supplemental Fig. 2). We speculate that there may be two reasons for this. First, the generation of cross adaption was sequentially regulated. Second, heat stress signal transduction may be partially independent to other stress. This result may also provide an experimental clue for study the mechanism of cross adaption in the future.

### Dynamic changes in transcriptional levels may be an important strategy for monocots and dicots to respond to short-term high-temperature stress

Compared to the WT, rice plants heterologously expressing *AtPLC9* exhibited a greater number of transcriptional changes, including changes in expression levels of TFs, when exposed to HS, which suggests that the regulation of gene transcription is an important strategy for plants to respond to short-term high-temperature stress.

We also observed abnormal expression of Ca^2+^-related signalling pathway genes in *AtPLC9*-expressing transgenic rice, suggesting that Ca^2+^ signalling may be an important pathway for plants to respond relatively quickly to short-term stress. Six marker genes were chose and the expressions were analyzed. These six genes response to environmental factors as reported in the plant Regulomics database (http://bioinfo.sibs.ac.cn/plant-regulomics/index.php) [38], an intergraded on-line database. The transcriptomic comparisons result indicated that the expressed levels of all these six genes can be regulated by biotic or abiotic stresses in previous studies. The result of study (GSE33204) indicated that *Os02g0527300* (*OsHsfA3*) and *Os03g0335200* (*OsWRKY44*) showed upregulation, and showed downregulation in 30-day-old leaves of rice after 6H high temperature treatment [39]. However, these three genes did not show alternated expression level in WT induced by heat stress in our study, which might be attributed to the different genotype, development stage, tissue or treatment condition. The expression of the remained genes can be induced or repressed by other biotic or abiotic stresses significantly. For example, significantly induced by salt treatment and highly expressed in spikelet and developing seeds, Os08g0540400 (OsCPK21) can improve the salt tolerance of rice [40]. Many factors affect the high-temperature stress response, beyond the modulation of gene expression. Therefore, it also may be interesting to look at the heat-tolerant phenotype induced by *AtPLC9* expression histologically.

### Feasibility of heterologous transformation for assessing gene function and conferring adaptive traits

Heterologous transformation helps the study of gene function and can be used to improve crop yield. This study demonstrated that AtPLC9 can also function in the monocotyledon rice. Heterologous transformation has broad applications in agricultural production, including the transfer of genes between wild and cultivated species, and between different families. However, researchers turning to heterologous transformation must consider the genetic diversity and evolutionary relationships between species. Heterologous genes may not retain their functions if transferred between species that have diverged too far.

We show that the heat-related gene *AtPLC9* from *Arabidopsis* functions well in rice in defending against heat shock. This contributes to our understanding of the molecular mechanisms of the heat-shock response in plants. Further exploration of AtPLC9 in other plant species could provide us more information regarding the functional conservation of elements in Ca^2+^/CaM HS signal transduction pathway. In addition, our results highlight that heterologous transformation has the potential to assist with molecular breeding both in monocots and dicots.

## Methods

### Plant materials and growth conditions

Rice seedlings were grown hydroponically in an illuminated incubator with 16 h light (28 °C) / 8 h dark (28 °C) photoperiod. Heat treatment was performed at 45 °C in a water bath. The seedlings grown in liquid medium for 7 days were moved in water bath for 45 °C HS treatment. Meanwhile, the control plants was put into water at 28 °C for same time. Cold treatment was performed at 4 °C in a low temperature incubator. For salt treatment, plants were exposed to 0 mM, 50 mM, 100 mM, 150 mM or 200 mM NaCl. To simulate drought, plants were exposed to 0 mM, 150 mM, 250 mM or 350 mM mannitol. Untreated seedlings were used as controls.

### Measurement of chlorophyll content and ion leakage

Chlorophyll content was determined as described by [41].

After HS treatment, 0.1 g seedlings was harvested in 4 ml deionized water. Conductivity of the solution was determined using a conductivity meter (METTLER TOLEDO FE38). Relative ion leakage was calculated according to the method of [25].

### Gene expression analysis

Total RNA was isolated from 14-day-old seedlings then translated into cDNA and used as template for quantitative PCR. *AtPLC9* transcript abundance in WT and transgenic plants was determined by RT-PCR using primers amplifying the *AtPLC9* coding region (Supplementary Table 2). *OsTUBULIN* was used as a control. Quantification of the expression level for AtPLC9-regulated genes in WT and OE-7 plants was performed by real-time quantitative PCR. Expression of *Os02g0781300*, *Os02g0527300*, *Os01g0968800*, *Os09g0401000*, *Os03g0335200*, *Os08g0540400* and *Os02g0781300* was analysed using primers given in Supplementary Table 2. Quantification of expression levels of *OsPLC* genes was also performed by real-time quantitative PCR using primers described by [16].

### RNA sequencing analysis

Total RNA was isolated from 14-day-old seedlings using a TRIzol kit (Invitrogen). Paired-end sequencing libraries with an average insert size of 200 bp were prepared with a TruSeq RNA Sample Preparation Kit version2 (Illumina) and sequenced on a HiSeq Xten according to the manufacturer’s standard protocols. FastQC (http://www.bioinformatics.babraham.ac.uk/projects/fastqc/) was initially run to assess the overall quality of all sequenced reads. Poor-quality reads were filtered out using Sickle with the parameters pe-mode; −t sanger–q 20 –l 50 (https://github.com/najoshi/sickle). The remaining high-quality reads were mapped to the *Oryza sativa Japonica* reference genome (http://plants.ensembl.org/Oryza_sativa/Info/Index) using TopHat v2.09 with the parameters “-N 3–read- edit-dist 3–segment-mismatches 1 -p 20 -r 0 -g 20 --microexon-search–b2-D 20–b2- R3–no-coverage-search”, and only reads showing unique alignments were retained for the following analysis. The cuffquant and cuffnorm components of cufflinks (2.2.1) with default parameters were used for normalization and estimation of gene expression levels. Only genes with a mean expression level of 1 read per kilobase of transcript per million mapped reads (FPKM) in at least one sample were considered expressed genes and used to calculate the Pearson correlation coefficient and PCA for replicates of each sample. HTseq software (http://www-huber.embl.de/users/anders/HTSeq/doc/overview.html) was used to count the read counts mapped to each of the genes. The Bioconductor package “edgeR” was used for differential expression analysis [42]. Only genes with FDR < 0.05 and absolute value of log2 (fold change) ≥ 1.0 were considered DEGs in subsequent analysis. GO term enrichment was analysed with Singular Enrichment Analysis provided by AgriGO [43] using a hypergeometric test, with FDR < 0.05 as cutoff. Rice GO terms downloaded from EnsemblPlants (http://plants.ensembl.org/index.html) were used as background references. The RNA-seq data were deposited in the NCBI SRA database (http://www.ncbi.nlm.nih.gov/sra) with BioProject number PRJNA597792.

## Supplementary Information

**Additional file 1: Supplemental Data Set 1**: List of genes differentially expressed between HS treatment and control of WT.

**Additional file 2: Supplemental Data Set 2**: List of genes differentially expressed between HS treatment and control of OE7.

**Additional file 3: Supplemental Data Set 3**: List of genes differentially expressed between OE7 and WT without HS treatment.

**Additional file 4: Supplemental Data Set 4**: List of genes differentially expressed between OE7 and WT after HS treatment.

**Additional file 5: Supplemental Data Set 5**: List and function annotation of putative targeted genes.

**Additional file 6: Supplemental Data Set 6**: Transcriptomic comparison results of six selected AtPLC9 targets generated from Plant Regulomics database.

**Additional file 7.**

## Data Availability

The datasets and material used and analyzed in this study are available from the corresponding authors on reasonable request.

## Abbreviations

HS: Heat shock
CDPKs: Calcium-dependent protein kinases
1,4,5-IP_3_: 1,4,5-inositol triphosphate
PLC: Phosphoinositide-specific phospholipase C
CaM: Calmodulin
