# Supplementary material for: Heterologous expression of heat stress-responsive AtPLC9 confers heat tolerance in transgenic rice
Source: BMC Plant Biol. 2020 Nov 11;20:514. doi: 10.1186/s12870-020-02709-5 (PMC7656764; doi:10.1186/s12870-020-02709-5)
Supplement: Supplementary file 7 — Additional file 7. [file 12870_2020_2709_MOESM7_ESM.zip › Supplemental Files.pdf]

Supplemental Figures and Tables

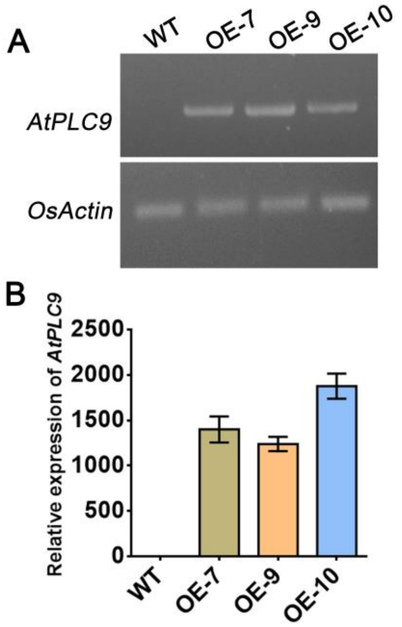

Supplemental Fig. 1. *AtPLC9* expression in WT and OE-7, OE-9 and OE-10 lines.
A. Semi-quantitative RT PCR to examine the *AtPLC9* transcript levels in wild type (WT) and AtPLC9-overexpression lines (OE-7, OE-9 and OE-10). *OsActin* was analyzed as the control.
B. Real-time PCR analysis of transcript levels of *AtPLC9*. *OsActin* was used as the internal reference. Error bars indicate mean ± SE from three independent experiments.

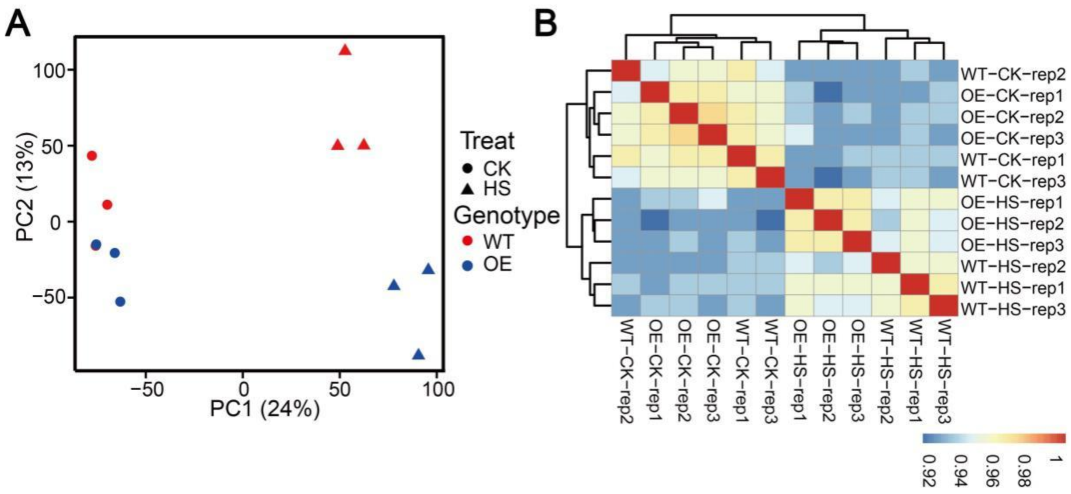

Supplemental Fig. 2. Global quality evaluation of RNA-seq data.
Four samples, WT-CK (wild type without HS), WT-HS (wild type after a 15-min HS), OE-CK (*AtPCL9*-expressing line 7 without HS) and OE-HS (*AtPCL9*-expressing line 7 after a 15-min HS), with three independent replicates were used for RNA-seq

analysis.
A. Principle component analysis (PCA) of all sequenced samples. Circles indicate control samples and triangles indicate HS-treated samples. Red and blue indicate wild-type (WT) and OE-7 lines, respectively.
B. Hierarchical cluster analysis of all RNA-seq data. The colour scale represents the Pearson Correlation Coefficient (PCC). The hierarchical clustering dendrogram was inferred by applying (1 − PCC) as distance function.
WT-CK and OE-CK indicate WT and OE-7 seedlings grown in Hoagland's solution under normal conditions (28 ℃) for 14 days, respectively. WT-HS and OE-HS indicate 14-day-old WT and OE-7 seedlings grown under normal conditions (28 ℃) treated at 45 ℃ for 15 min.

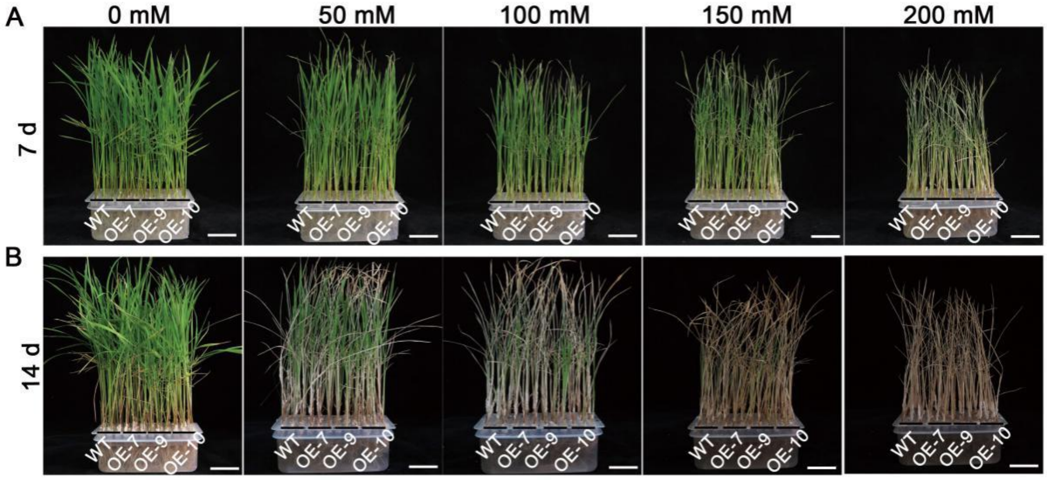

Supplemental Fig. 3. Expression of *AtPLC9* in rice does not affect salt sensitivity. WT and transgenic lines OE-7, OE-9, OE-10 were grown in Hoagland's solution under normal conditions for 7 days before addition of 0, 50, 100, 150 or 200 mM sodium chloride for another 7 (A) or 14 (B) days. Bars = 3 cm.

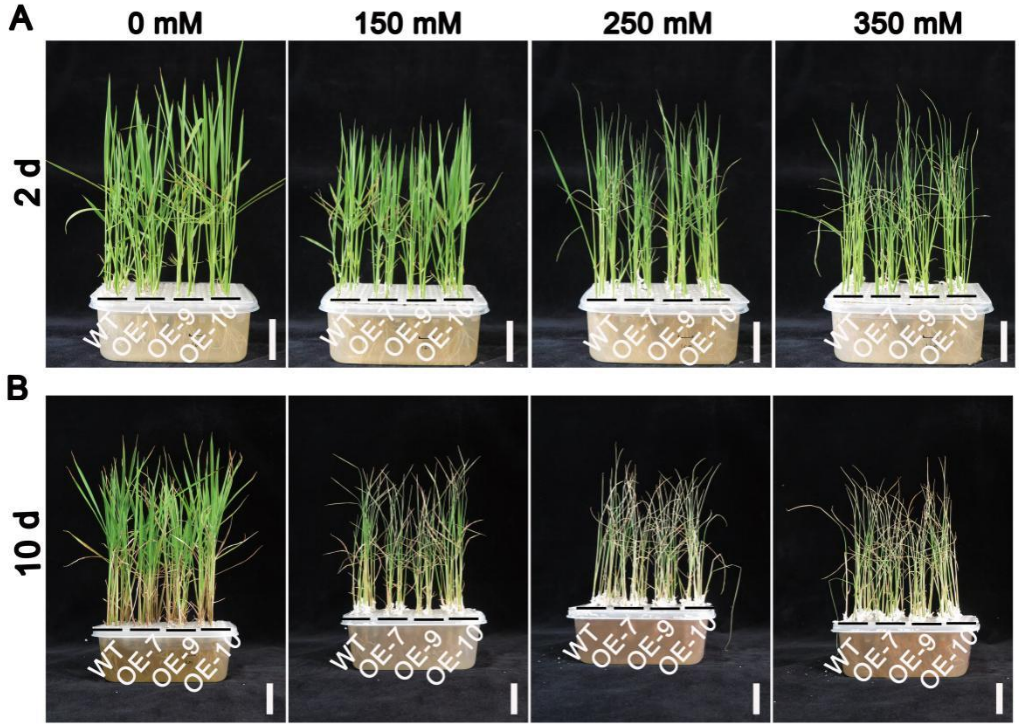

Supplementary Fig. 4. Expression of *AtPLC9* in rice does not affect drought susceptibility.

A, WT and transgenic lines OE-7, OE-9 and OE-10 were grown in Hoagland's solution under normal conditions for 7 days before the addition of 0, 150, 250 or 350 mM mannitol for a further 2 (A) or 10 (B) days. Bars = 3 cm.

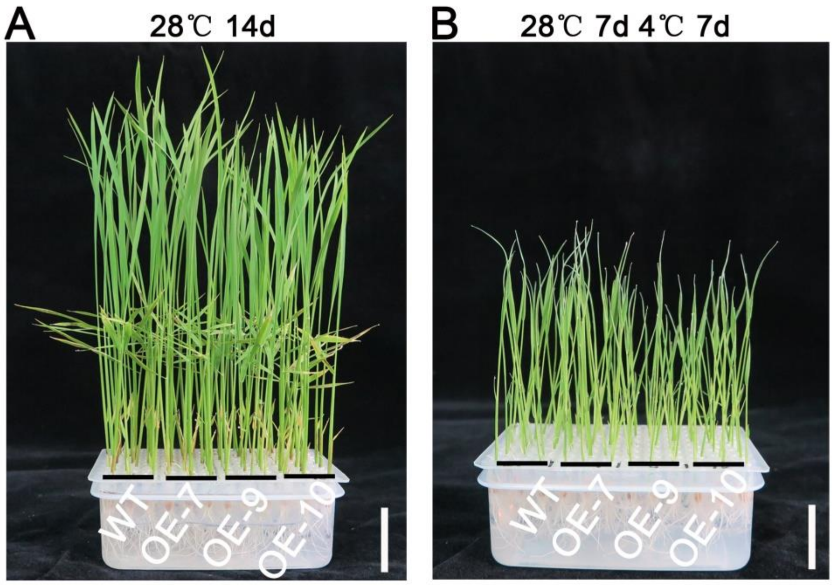

Supplementary Fig. 5. Expression of *AtPLC9* in rice does not affect sensitivity to low temperature.

A, Fourteen-day-old seedlings (WT, OE-7, OE-9 and OE-10) grown in Hoagland’s solution under 28 ℃ conditions.

B, Seven-day-old seedlings (WT, OE-7, OE-9 and OE-10) grown in Hoagland’s solution under 28 ℃ conditions were shifted to 4 ℃ for 7 days and then returned to normal conditions for 7 days to observe phenotypes. Bars = 3 cm.

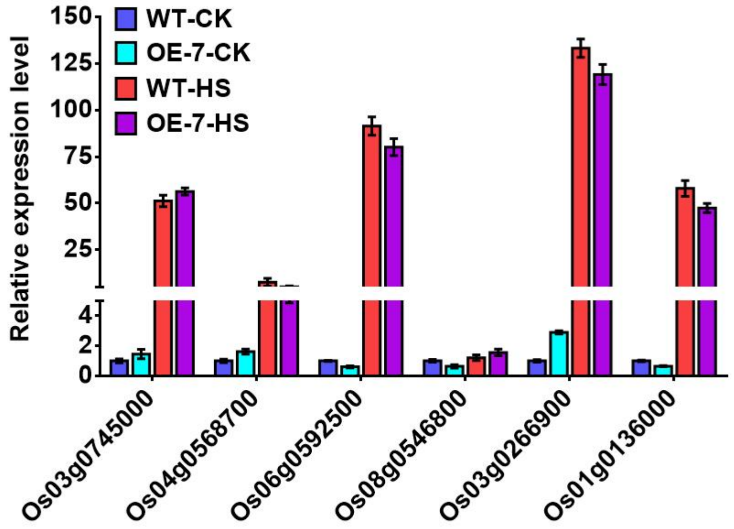

Supplementary Fig. 6. Expression of genes for HSFs (*Os03g0745000*, *Os04g0568700*, *Os08g0546800*), Multiprotein-bridging factor 1c (MBF1 *Os06g0592500*) and HSPs (*OS01g0136000* and *Os03g0266900*) in wild-type (WT) and OE-7 plants before (CK) and after HS treatment. Values for Realtime-PCR results are means ± SE (n = 3).

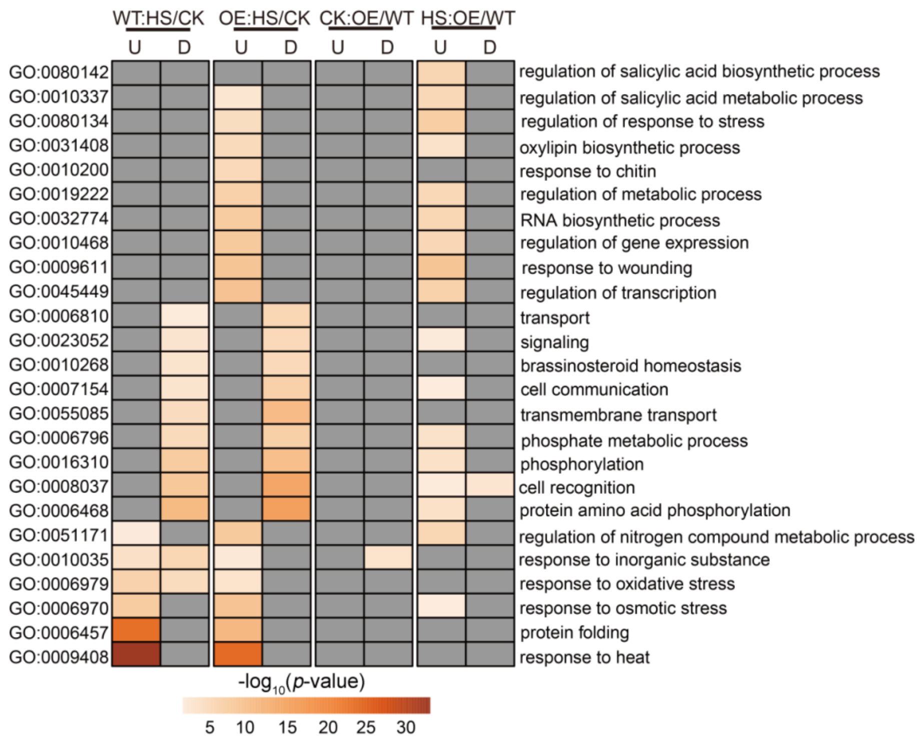

Supplementary Fig. 7. GO functional categories of genes with transcriptionally up- and downregulated levels for the indicated comparisons. The color in each cell indicates −log10 (P values) of the GO enrichment according to the scale shown; grey cells are not significant. U, upregulation; D, down regulation.

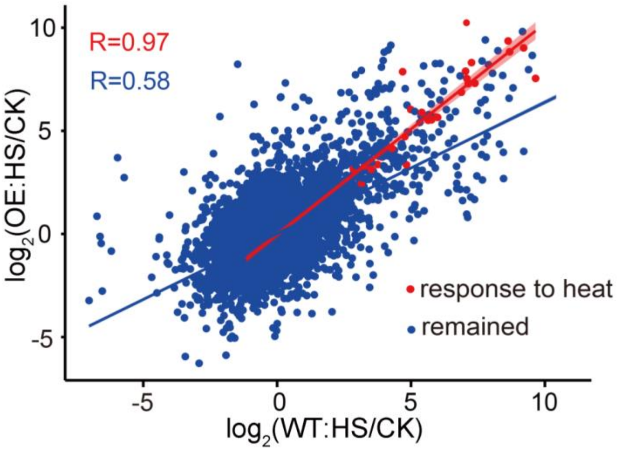

Supplementary Fig. 8. Scatterplot showing the correlation of log2-transformed fold change of expression level after HS between wild type (x axis) and OE-7 (y axis). Red and blue dots represent genes with GO term ‘response to heat’ (GO:0009408) and the remaining genes, respectively.

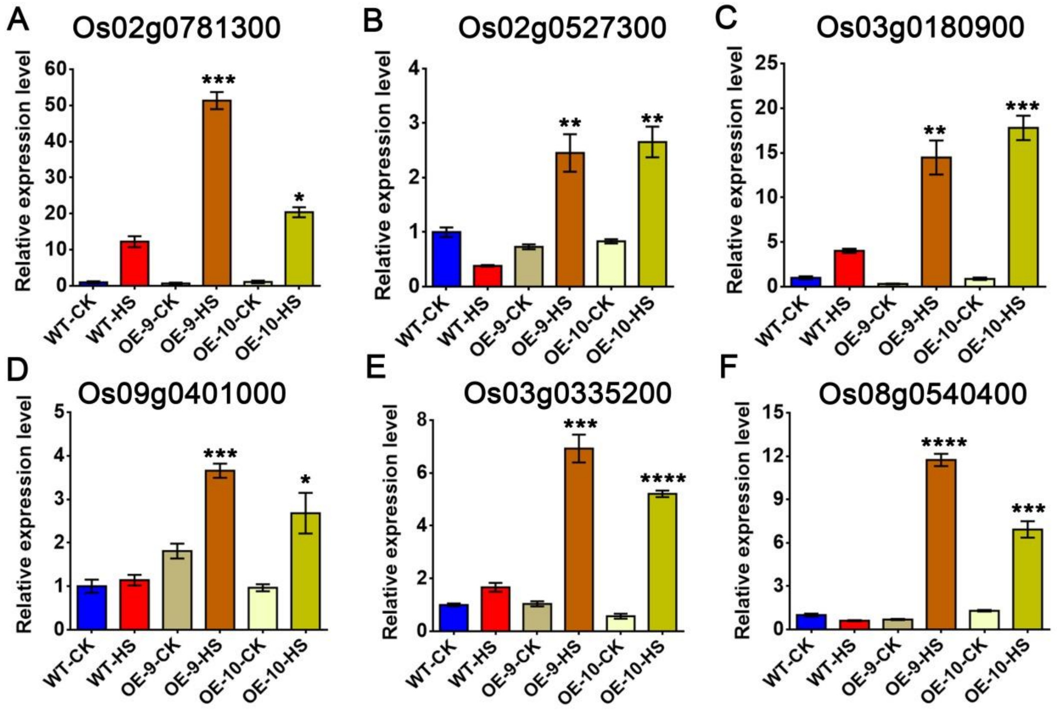

Supplementary Fig. 9. Validation of RNA-seq data using real-time quantitative PCR for six AtPLC9 target genes. Real-time quantitative PCR quantification of RNA levels for six candidate AtPLC9-regulated genes (A–F) in wild-type (WT) and OE-9 and OE-10 plants before (CK) and after HS treatment. Values for real-time quantitative PCR results are means ± SE (n = 3). Student's t-test was used to calculate P values. ****P value <0.0001, ***P value <0.001, **P value <0.01, *P value <0.05 compared to WT-HS.

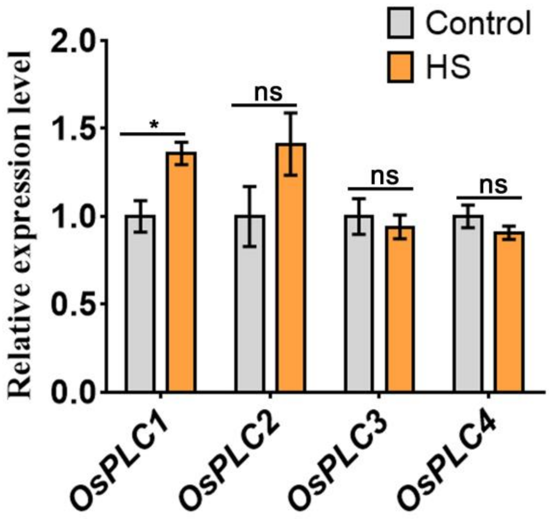

Supplementary Fig. 10. Endogenous *OsPLC* gene expression remains unchanged after HS treatment.

Fourteen-day-old WT seedlings grown in Hoagland's solution under 28 ℃ conditions were shifted to 45 ℃ for 15 min for HS treatment; seedlings without HS were used as a control. Total RNA was extracted for real-time quantitative PCR analyses of *OsPLC*

gene expression. Expression levels were normalized to those of *ACTIN*. Each value is the mean ± SE, $n = 3$. Similar results were obtained from three independent experiments. Student's *t*-test was used to calculate *P* values. *P value <0.05 compared to HS. ns, not significant.

Supplementary Table 1. Statistics of RNA-seq data and read mapping

Library	Total number of reads	High-quality reads		Mapped reads		Uniquely mapped reads	
		Number	Percentage	Number	Percentage	Number	Percentage
WT-CK-rep1	67,192,744	66,246,782	98.59%	59,225,741	88.14%	49,287,164	73.35%
WT-CK-rep2	78,388,842	77,751,218	99.19%	70,967,829	90.53%	60,781,585	77.54%
WT-CK-rep3	85,288,462	84,249,062	98.78%	75,516,253	88.54%	65,485,080	76.78%
WT-HS-rep1	65,789,934	65,121,886	98.98%	59,392,644	90.28%	49,916,736	75.87%
WT-HS-rep2	64,867,808	64,343,140	99.19%	59,046,310	91.03%	49,815,460	76.80%
WT-HS-rep3	64,971,592	63,604,556	97.90%	58,467,462	89.99%	50,786,971	78.17%
OE-CK-rep1	70,087,460	68,723,290	98.05%	62,770,349	89.56%	54,964,716	78.42%
OE-CK-rep2	78,159,638	77,363,778	98.98%	70,947,286	90.77%	60,920,415	77.94%
OE-CK-rep3	81,893,780	80,644,728	98.47%	73,423,318	89.66%	63,077,705	77.02%
OE-HS-rep1	78,548,050	77,803,226	99.05%	69,804,842	88.87%	57,639,139	73.38%
OE-HS-rep2	79,513,880	78,730,638	99.01%	71,271,662	89.63%	60,961,342	76.67%
OE-HS-rep3	65,142,166	64,231,014	98.60%	57,765,326	88.68%	50,315,755	77.24%

Supplementary Table 2. Primer sequences

Primer name	Sequence	Gene
FP1	5′- ACGGAGCTCAATCCCCCGATCAGT -3′	*AtPLC9* RT-PCR
RP1	5′- GCATCAACCAAAGTTTTTCTTTGT -3′	
FP2	5′- AGGAAGGCTGGAAGAGGACC -3′	*OsActin*
RP2	5′- CGGGAAATTGTGAGGGACAT -3′	
FP3	5′- GCCATGCCTACGACTTCTTG -3	Os02g0781300
RP3	5′-TAGGAGTCTCGCGTGTCAAA-3′	
FP4	5′-AGGTCCTCACCAACACAACA-3′	Os02g0527300
RP4	5′-CCTCCTGAAGCAATGCACTC-3′	
FP5	5′-GAGGACGCCATCTTCGACAT -3′	Os01g0968800
RP5	5′-TCGAGAGATCTCCCAATCGC -3′	
FP6	5′-ACTGGAACACGCACATCAAG-3′	Os09g0401000
RP6	5′-GACGACTTCTCCTCCACCTC-3′	
FP7	5′-CAGGGACCCTTCCACGATTA-3′	Os03g0335200
RP7	5′-GGCGAAGCTGATGATGTTGT-3′	
FP8	5′-CAGGAGGCTCTGCAAGGATA-3′	Os08g0540400
RP8	5′-ATCCCTGTCTGGTGAAGGTG-3′	
FP9	5′-ACACCTACGGGTTCAGGAAG-3′	Os03g0745000
RP9	5′-TGGTGCTGCGACGACGACGA-3′	
FP10	5′-CCTACGGATTCAAGAAGGTGGT-3′	Os04g0568700
RP10	5′-ATCGGTATCGCCGTCGGTATGC-3′	
FP11	5′-GAGGTTGAGCGGCAACATCA-3′	Os06g39240

RP11	5′-GTCCGCATCGCCTGGTTCAC-3′	
FP12	5′-CAGCTCAACACCTAGGATTTAG-3′	Os08g0546800
RP12	5′-AGCTGGAGGATATGACCTGC-3′	
FP13	5′-AGGTCAAGGTGGAGGTTGAG-3′	Os03g0266900
RP13	5′-CCACTTGTCCGTCTTCTCCT-3′	
FP14	5′-CAGCAAGGAGAAGGAGGACA-3′	Os01g0136000
RP14	5′-GCTTCTTGACCTCAGCCTTG-3′	
FP15	5′-TGCACGGTGCACAAATGATT -3′	*AtPLC9* Realtime-PCR
RP15	5′-GACGTAACCGCACCCTCCAT-3′	
