# Supplementary figures and images for: Heterologous expression of heat stress-responsive AtPLC9 confers heat tolerance in transgenic rice
Source: BMC Plant Biol. 2020 Nov 11;20:514. doi: 10.1186/s12870-020-02709-5 (PMC7656764; doi:10.1186/s12870-020-02709-5)

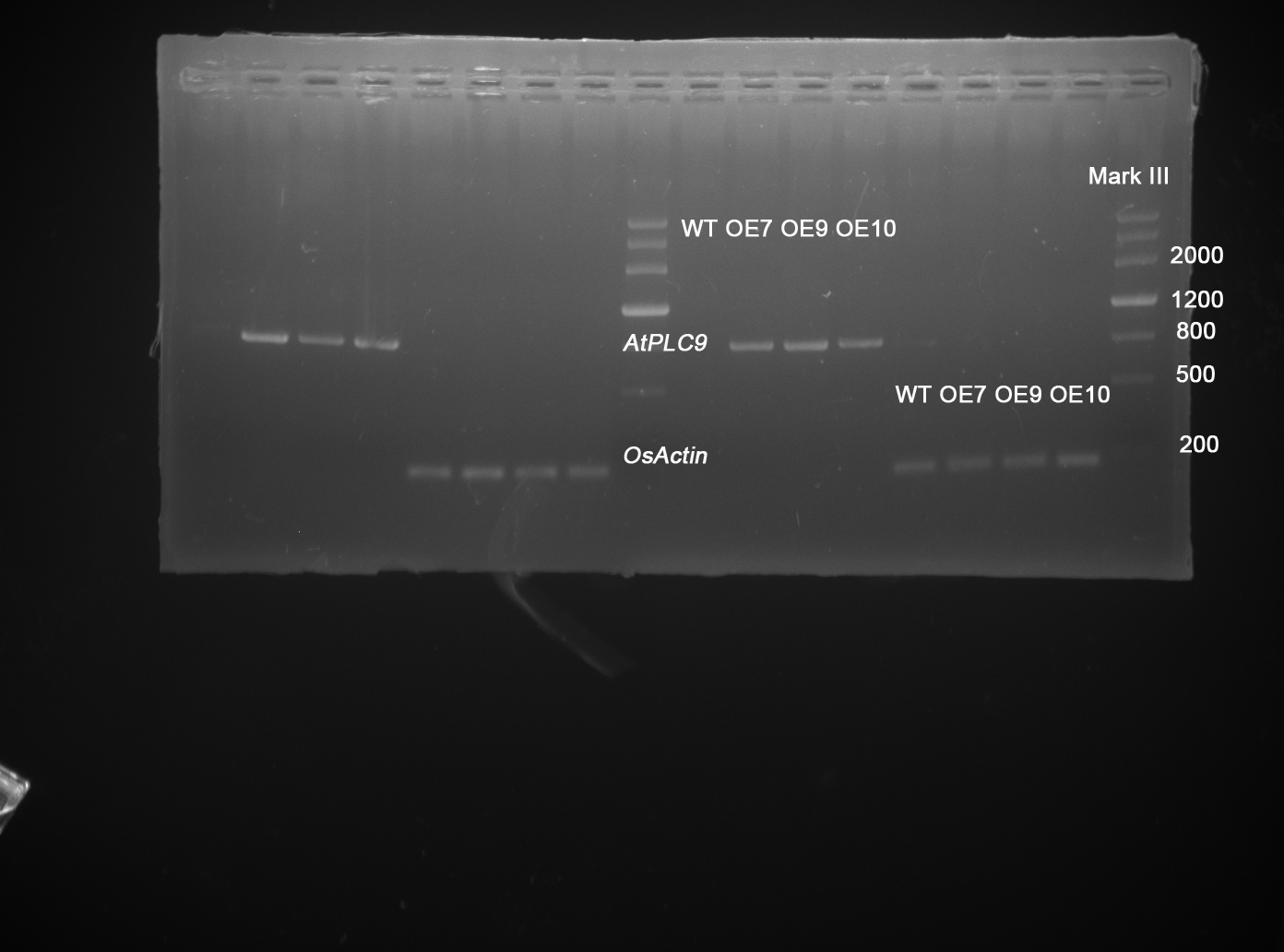

Supplement: Supplementary file 7 — Additional file 7. [file 12870_2020_2709_MOESM7_ESM.zip › Fig S1-Original image.tif]

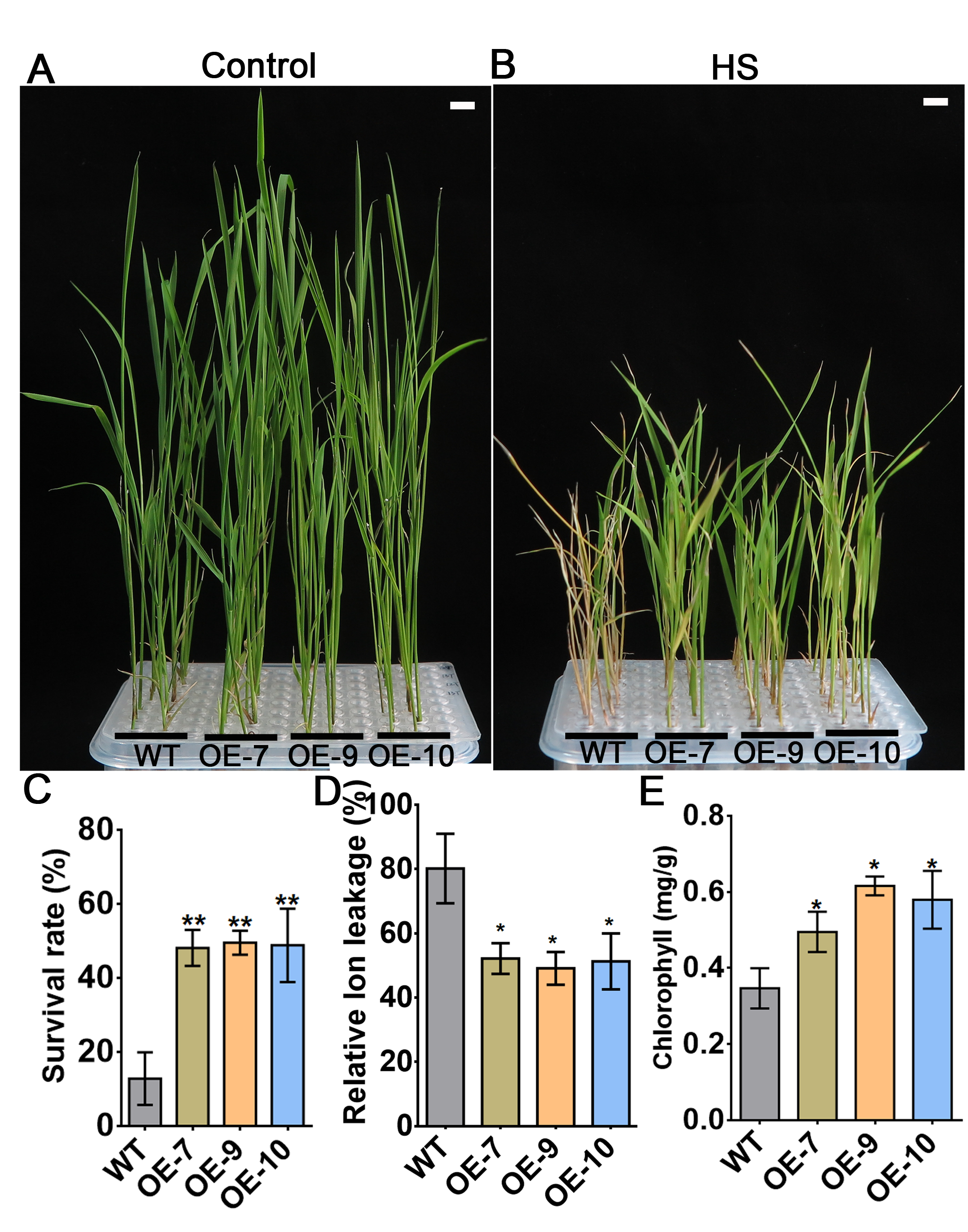

Supplement: Supplementary file 7 — Additional file 7. [file 12870_2020_2709_MOESM7_ESM.zip › Supplemental Fig2.tif]
